# Photocatalytic removal of imidacloprid containing frequently applied insecticide in agriculture industry using Co_3_O_4_ modified MoO_3_ composites

**DOI:** 10.3389/fchem.2023.1125835

**Published:** 2023-03-14

**Authors:** Mohamed Shaker S. Adam, Sumbleen Sikander, M. Tariq Qamar, Shahid Iqbal, Ahmed Khalil, Amel Musa Taha, Obadah S. Abdel-Rahman, Eslam B. Elkaeed

**Affiliations:** ^1^ Department of Chemistry, College of Science, King Faisal University, Al-Ahsa, Saudi Arabia; ^2^ Department of Chemistry, Faculty of Science, Sohag University, Sohag, Egypt; ^3^ Department of Chemistry, Forman Christian College (A Chartered University), Lahore, Pakistan; ^4^ Department of Chemistry, School of Natural Sciences (SNS), National University of Science and Technology (NUST), Islamabad, Pakistan; ^5^ Chemistry Department, Faculty of Science, Zagazig University, Zagazig, Egypt; ^6^ Department of Pharmaceutical Sciences, College of Pharmacy, AlMaarefa University, Riyadh, Saudi Arabia

**Keywords:** insecticide, imidacloprid, greeda, MoO3 composites, photocatalysis

## Abstract

Water pollution caused by the frequent utilization of pesticides in the agriculture industry is one of the major environmental concerns that require proper attention. In this context, the photocatalytic removal of pesticides from contaminated water in the presence of metallic oxide photocatalysts is quite in approach. In the present study, Orthorhombic MoO_3_ has been modified with varying amount of cobalt oxide through wet impregnation for the removal of imidacloprid and imidacloprid-containing commercially available insecticide. The solid-state absorption response and band gap evaluation of synthesized composites revealed a significant extension of absorption cross-section and absorption edge in the visible region of the light spectrum than pristine MoO_3_. The indirect band gap energy varied from ∼2.88 eV (MoO_3_) to ∼2.15 eV (10% Co_3_O_4_-MoO_3_). The role of Co_3_O_4_ in minimizing the photo-excitons’ recombination in MoO_3_ was studied using photoluminescence spectroscopy. The orthorhombic shape of MoO_3_ was confirmed through X-ray diffraction analysis and scanning electron microscopy. Moreover, the presence of distinct absorption edges and diffraction peaks corresponding to Co_3_O_4_ and MoO_3_ in absorption spectra and XRD patterns, respectively verified the composite nature of 10% Co_3_O_4_-MoO_3_. The photocatalytic study under natural sunlight irradiation showed higher photocatalytic removal (∼98%) of imidacloprid with relatively higher rate by 10% Co_3_O_4_-MoO_3_ composite among all contestants. Furthermore, the photocatalytic removal (∼93%) of commercially applied insecticide, i.e., Greeda was also explored.

## Introduction

With the arrival of the Green Revolution in the 20^th^ century, farmers started to use pesticides, insecticides, and herbicides to get higher yields. These chemicals are frequently applied by farmers not only in crops and farms but also in backyard gardens to prevent the agriculture field from the pest attack. The use of pesticides and insecticides at various stages has significantly increased food production and made food storage safer (Foster et al., 1991; Mateo-Sagasta et al., 2017). However, the excessive use of these toxins causes many adverse effects such as pollution of brooks, rivers, marshy areas, lakes, and also valleys, as these toxic manufactured chemicals run off into the nearby water streams which ultimately leads to the scarcity of clean water (Kaur et al., 2019). It is reported that the world is utilizing approximately 2.5 million tons of pesticides on an annual basis wherein very small quantities reach the target while the rest disperse through air, soil, and water which consequently leads to pollution (Hodgson and Levi, 1996).

Many concerns are shown by researchers nowadays because of the permanent existence of pesticides and insecticides in water and food as they also have inauspicious effects on human health and the equilibrium of the ecosystem (Carvalho, 2017; de Souza et al., 2020). Human health is badly affected by these toxins in the drinking water. These toxins have critical effects on mortals such as carcinogenesis, neurotoxicity effects on reproduction, and cell development effects, commonly in the early stages of life (Villaverde et al., 2017; Liang et al., 2019). Their effects are equally harmful to human beings, animals, and marine life. The situation is much critical in countries where water resources are limited, and lives are restricted to utilize contaminated water for various purposes. Therefore, it is an acute need to come up with an efficient and cost-effective technology for the removal of these toxins from contaminated water (Ahmed et al., 2011; Khan et al., 2015a).

Several water decontamination techniques have been utilized to remove the pollutants from the contaminated water such as adsorption, filtration, chemical oxidation, and biological treatments. In addition, many studies are available for the utilization of advanced oxidation methodologies such as photocatalytic process, heterogeneous catalytic oxidation with H_2_O_2_, Fenton/photo-Fenton oxidation, ozonation, and UV/H_2_O_2_ treatment for water purification (Swaminathan et al., 2013; Cardoso et al., 2021). Among these, heterogeneous photocatalysis for the complete removal and mineralization of organic toxins is becoming advantageous over others due to its reusability, recovery of photocatalysts, suitability, and sustainability (Qamar et al., 2017; Alhogbi et al., 2020; Aslam et al., 2022). Moreover, it is considered as a green process because natural sunlight can be used to initiate a photocatalytic process while generating reactive oxygen species (ROS) which ultimately leads to the conversion of pollutants into benign species (Zangeneh et al., 2015; Charanpahari et al., 2019).

The progress of photocatalysis is significantly based on the choice of a photocatalyst. In this context, metal oxides and metal oxide-based photocatalysts are being studied extensively for the removal of toxins due to their tunable morphological, structural, optical and photocatalytic properties (Mathiarasu et al., 2021; Hannachi et al., 2022a; Maqbool et al., 2022). Recently, ternary composite of MoO_3_ with CuO, MoO_3_/ZnO heterostructures, CuO/MoO_3_ heterojunction, ZnO co-doped with Ce and Yb and MoO_3_/g-C_3_N_4_ have been used as photocatalysts wherein a synergic effect of the components in a composite was considered responsible for the enhanced photocatalytic removal of organic dyes (Xue et al., 2019; Ramesh and Shivanna, 2021; Hannachi et al., 2022b; Hussain et al., 2022). In addition, many investigations related to the photocatalytic activities of TiO_2_, ZnO, Fe_2_O_3_, WO_3_, Bi_2_O_3_, V_2_O_5_, Cu_2_O, NiO, *etc.*, Have also been reported (Khan et al., 2015b; Mathiarasu et al., 2021; Velempini et al., 2021; Hannachi et al., 2022a; Maqbool et al., 2022). However, the quest to investigate the potential contestant for the effective removal of toxic pollutants from the wastewater has not over yet and researchers are also paying attention to study the photocatalytic removal of pesticides and insecticides from the aqueous system in the illumination of light.

For the above-mentioned purpose, Molybdenum trioxide (MoO_3_), an n-type semiconductor, can be an interesting candidate because of having wide band gap energy, ionic conductivity as a result of oxygen vacancies, layered structure, tunable optoelectronic properties and photocatalytic activity (Ijeh et al., 2020; Ali and Ahmad, 2022). Due to its enthralling electrical and optical properties, it is being used in various applications such as gas sensing, LED, smart windows, supercapacitors, batteries, energy storage, DSSCs, optical switch coatings and photocatalysis (Al-Muntaser et al., 2022; Noby et al., 2022; Selvakumar and Palanivel, 2022). However, its wide application in photocatalytic removal of organic toxins is limited due to its large band gap (∼3.1 eV), smaller visible-light absorption cross-section and lower photo-excitons separation (Zheng et al., 2021).

In the above contexts, this study has been designed to address the two issues; Firstly, orthorhombic MoO_3_ has been modified with Co_3_O_4_ through the wet-impregnation method to come up with a photocatalyst having larger light absorption spectrum, lower band gap energy and greater charge separation and transferability. Secondly, the successful removal of 15 ppm imidacloprid and imidacloprid containing commercially applied insecticide (Greeda) in the agriculture field under the exposure of natural sunlight. The reason for the utilization of Co_3_O_4_ to modify n-type MoO_3_ is due to its light absorption capacity, narrow band gap, good charge transportability and p-type semiconducting nature (Cao et al., 2022; Wang et al., 2023). Moreover, the successful contribution of Co_3_O_4_ in enhancing the photocatalytic activity of CeO_2_, g-C_3_N_4_, TiO_2_, Bi_2_O_3_ and Fe_2_O_3_ while generating photo-excitons’ separation is well established (Ranjith et al., 2019; Niu et al., 2022; Reena et al., 2022). Previously, rare studies are available for the removal of imidacloprid over MoO_3_-based photocatalysts (Adabavazeh et al., 2021; Zhang et al., 2021). However, there is no investigation is present yet for the removal of imidacloprid and Greeda over Co_3_O_4_-modified orthorhombic MoO_3_ under the illumination of natural sunlight.

## Experimental

Orthorhombic MoO_3_ was prepared using co-precipitation method. In this context, 12.35 g (NH_4_)_6_Mo7O24.4H2O (Sigma-Aldrich, ≥99%) was dissolved in distilled water with continuous stirring for an hour at room temperature. Then 2 mL Triton X-100 (Sigma-Aldrich, laboratory-grade) was added to the clear solution. The mixture was then acidified with slow addition of 0.1 M HNO_3_ (Sigma-Aldrich, 70%) to attain the pH of the solution to 2 with constant heating at 50°C till the formation of precipitates. The precipitates were separated from the mixture through filtration, washed, and dried in an oven at 100°C. The dried precipitates were calcined at 500°C for 4 h at 10°C/min in a Vulcan D-550 muffle furnace. The calcined material was ground using mortar and pestle to get powder of α-MoO_3_ photocatalyst.

**SCHEME 1:**
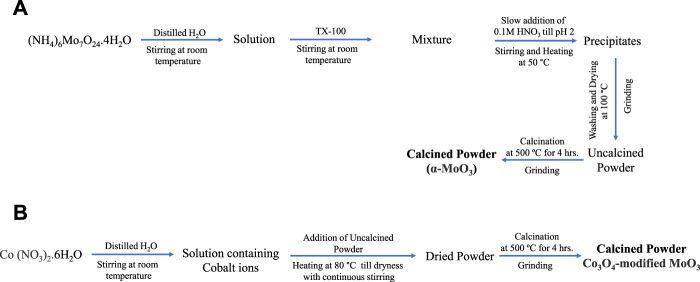
The schematic presentation for the synthesis of **(A)** α-MoO_3_ and **(B)** Co_3_O_4_-modified MoO_3_ powders.

The Co_3_O_4_-modified MoO_3_ was synthesized using the wet-impregnation method (Scheme 1). The composite of 1 wt% Co_3_O_4_-MoO_3_ was prepared by dissolving 0.15 g Co (NO_3_)_2_.6H_2_O (Sigma-Aldrich, ≥99%) in distilled water in a beaker (100 mL) with continuous stirring at room temperature. Then, 3.0 g pre-synthesized α-MoO_3_ was added to the cobalt nitrate solution under continuous stirring at 80°C. The mixture was kept on a hot plate till complete dryness at 80°C. The dried material was collected and calcined at 500°C for 4 h at 10°C/min in a Vulcan D-550 muffle furnace. The calcined material was ground using mortar and pestle to get powder of 1 wt% Co_3_O_4_-MoO_3_ photocatalyst. Moreover, a similar procedure was adopted to synthesize 3, 5, and 10 wt% Co_3_O_4_-MoO_3_ by using 0.45, 0.75 and 1.5 g Co (NO_3_)_2_.6H_2_O, respectively.

The synthesized materials were characterized using optical, structural and morphological techniques. The solid-state absorption spectra were recorded in the 200–850 nm range using Perkin Elmer UV-visible diffuse reflectance spectrophotometer whereas the band gap energy of the synthesized materials was estimated using Kubelka- Munk, *F(R),* the transformation of reflectance (%). The change in photo-excitons recombination was noticed from the photoluminescence response of the materials acquired from fluorescence spectrometer, RF-5301 PC, Shimadzu, Japan, at 200 nm excitation wavelength. The XRD patterns were recorded from 5 to 90 with 0.02° step size by X’pert X-ray powder diffractometer (Philips PW1398) having Cu Kα radiation source. Moreover, the morphology of the 10% Co_3_O_4_-MoO_3_ was examined by field-emission scanning electron microscopy (Hitachi, SU8010, Tokyo, Japan).

The photocatalytic removal of 15 ppm imidacloprid (Merck, Analytical standard) was studied under the illumination of natural sunlight (900 ± 50 × 10^2^ lx). In a typical experiment, the optimized dose of pristine MoO_3_ (150 mg) was suspended in 100 mL 15 ppm imidacloprid aqueous solution and stirred for 10 min. The suspension was poured into a glass reactor of 14 cm (diameter) and 2 cm (height) and kept in dark for 30 min. After 30 min, the glass reactor was exposed to natural sunlight. The samples were collected after a regular time interval (light exposure) of 30 min and filtered using a 0.20 µm syringe filter. The filtered samples were then subjected to a UV-visible spectrophotometer (UV-1800, Shimadzu, Japan) for the monitoring of imidacloprid removal while selecting its absorbance at a characteristic wavelength, i.e., 270 nm (λ_max_) (Kanwal et al., 2018). The same procedure was adopted to monitor the removal of 15 ppm imidacloprid over other synthesized Co_3_O_4_-modified MoO_3_ composites.

In order to study the removal of imidacloprid from the polluted water, the samples were collected using a syringe filter from the suspension exposed to the light after a regular interval and were subjected to UV-visible spectrophotometer for the determination of the concentration of removed pollutant which ultimately led to the % removal of imidacloprid by the photocatalyst under the exposure of light using the following equation (Garg et al., 2021).

$$\%\;removal=\frac{C_{o}-C_{t}}{C_{o}}\times 100$$ (1)

Here, *C*_*o*_ is the pollutant initial concentration whereas *C*_*t*_ is the concentration of the pollutant after exposure time *‘t'*. Moreover, the validity of Langmuir Hinshelwood (L-H) kinetic models was also studied using the following equation to evaluate the kinetics of photocatalytic removal of organic pollutants under the exposure of light (Alhogbi et al., 2020).

$$\ln\frac{C_{o}}{C_{t}}=k\times t$$ (2)

Here, *k* is the rate constant which was determined from the slope by plotting *ln* $\frac{C_{o}}{C_{t}}$ V *t*.

Moreover, the photocatalytic removal of 15 ppm Greeda (Agricom international, Jiangsu Pesticide Research Institute, Jiangsu, China), a commercially available insecticide in the agriculture field, over 10% Co_3_O_4_-MoO_3_ was also investigated under the illumination of natural sunlight.

## Results and discussion

**FIGURE 1:**
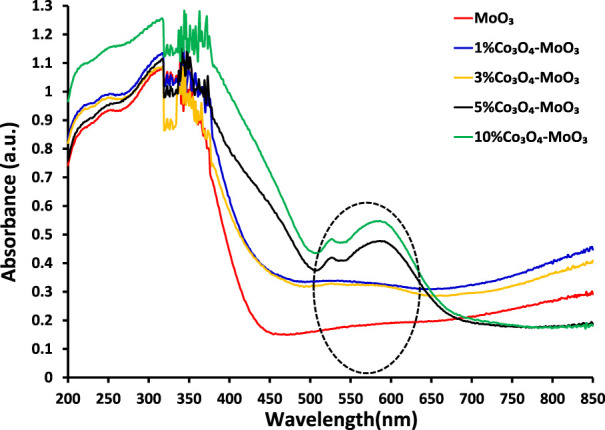
The comparison of solid-state absorption spectra of pristine and modified MoO_3_.

**FIGURE 2:**
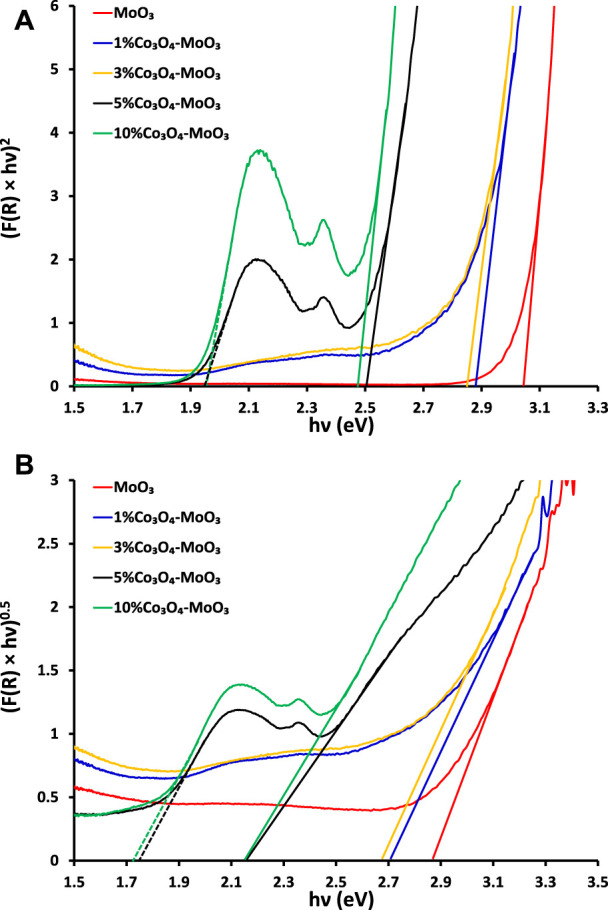
The graphical evaluation of **(A)** direct **(B)** indirect band gaps of pristine and modified MoO_3_.

**SCHEME 2:**
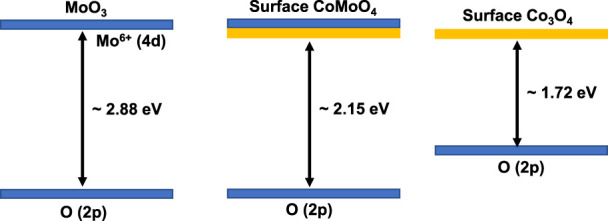
The representation of band gaps in synthesized materials.

The comparison of solid-state absorption spectra of MoO3 and its composites is presented in Figure 1 Which indicates the beneficial role of Co3O4 in increasing the absorption cross-section of MoO3. Wherein, maximum effect in enhancing the absorption response was noticed for 10% Co3O4-MoO3 composite. Moreover, the presence of distinct absorption due to Co3O4 (encircled in Figure 1) in addition to the major absorption corresponding to MoO3 confirms the composite nature of the synthesized materials. Band gap energy of the synthesized materials has been estimated by plotting (*F(R) × hν*)^1/n^ *versus* hυ (eV). Here, “n” can be ½ or two which corresponds to direct or indirect band gaps evaluation, respectively and F(R) is Kubelka−Munk function. The direct and indirect band gap evaluations (Figures 2A,B) reveal a lowering in the band gap energy of composite materials as compared to pristine MoO_3_. The evaluated indirect band gap (∼2.88 eV), as shown in Figure 2B has close agreement with literature values for MoO_3_ which is mainly due to the excitations of the electron from O (2p) to Mo (4days) (Qamar et al., 2017). Whereas, the decrease in band gap energy of the composite than pristine MoO_3_ is attributed to the introduction of cobalt (3days-4s) orbitals below in the vicinity of the MoO_3_ conduction band as shown in Scheme 2. The indirect band gap energies, estimated by extrapolating (*F(R) × hν*)^*0.5*^ vs*. hν* curves to the horizontal axis were ∼2.70 eV, ∼2.68 eV, ∼2.16 eV and ∼2.15 eV for 1%, 3%, 5% and 10% Co_3_O_4_-MoO_3_, respectively as shown in Figure 2B. The prominent multiple band edge near ∼1.7 eV was also noticed at higher loading (5% and 10%) of Co_3_O_4_ in composites, which is in accordance with band gap energy of Co_3_O_4_ presented in literature (Bhargava et al., 2018; Anuradha and Raji, 2020). Hence, the presence of these multiple edges in modified MoO_3_ confirms the composite nature of the synthesized photocatalyst.

**FIGURE 3:**
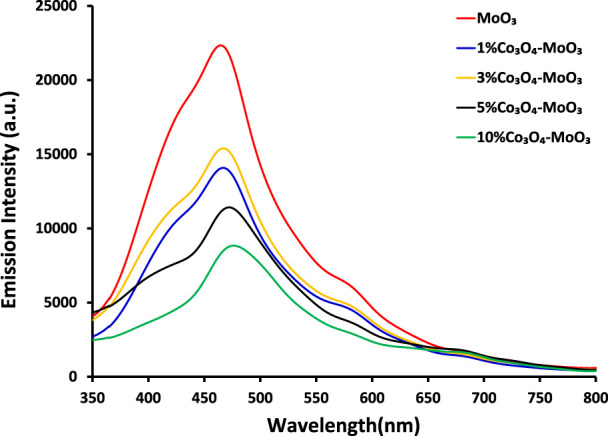
The comparison of photoluminescence spectra of pristine and modified MoO_3_.

Figure 3 depicts the comparison of photoluminescence spectra of synthesized photocatalysts, where a successive decrease in emission intensity was noticed for all composites as compared to pristine MoO_3_ indicating the supporting role of Co_3_O_4_ in lowering the recombination process. Among composites, 10% Co_3_O_4_-MoO_3_ showed the smallest photo-excitons (*e-–h+*) recombination which favors the fruitful use of photo-excitons in photocatalytic removal of target pollutant, i.e., insecticide. Moreover, a ∼60% decrease in emission intensity was observed for 10% Co_3_O_4_-MoO_3_ whereas ∼31, ∼3 seven and ∼49% decrease in emission intensity was noticed for 1, 3, and 5% Co_3_O_4_-MoO_3_ composites, respectively.

**FIGURE 4:**
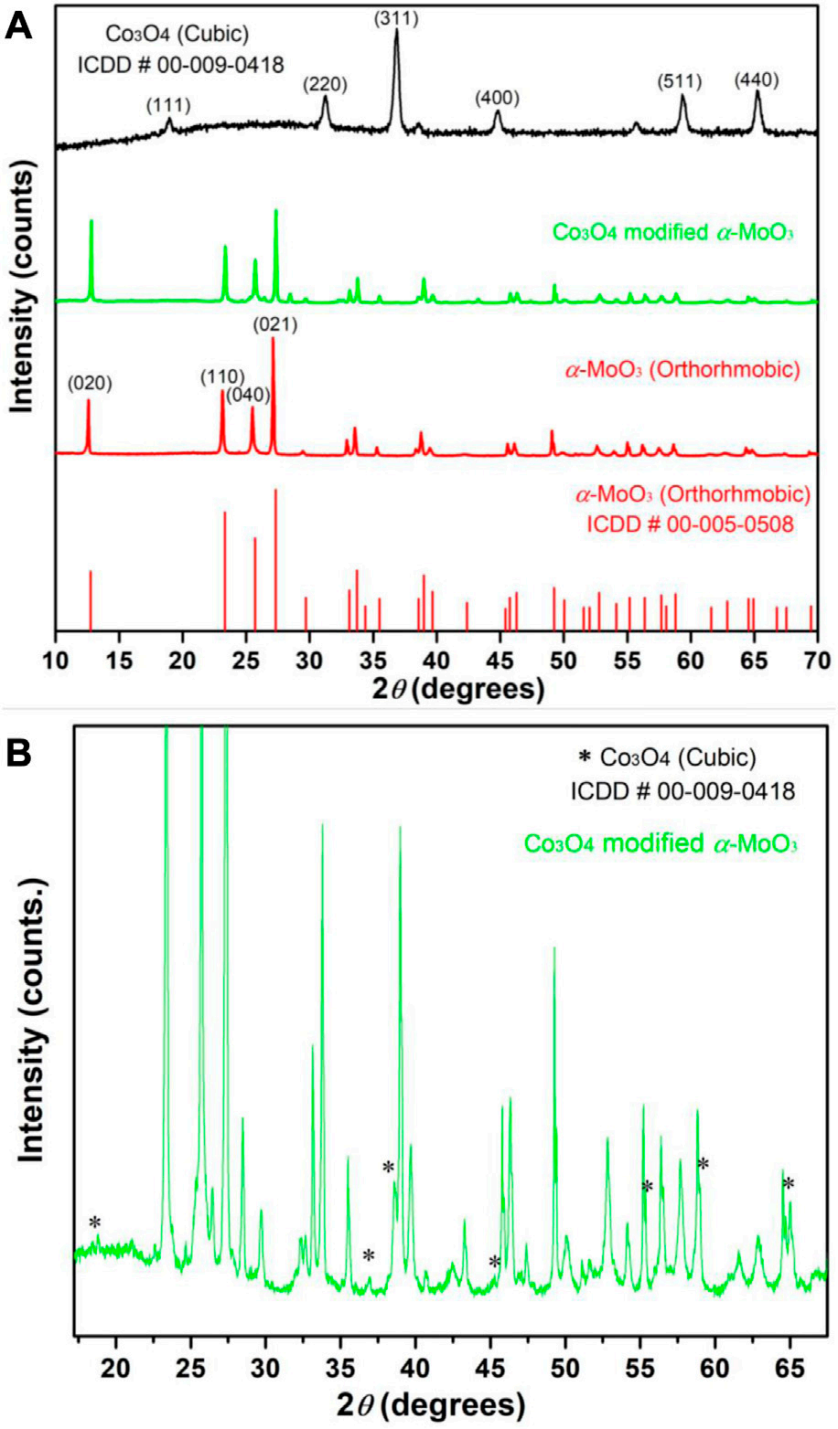
The XRD patterns of **(A)** α-MoO_3_, Co_3_O_4_ and 10% Co_3_O_4_ modified MoO_3_ whereas **(B)** is the exploded XRD pattern of 10% Co_3_O_4_ modified MoO_3_ with clearly marked (*) reflections by Co_3_O_4_.

The comparison of x-ray diffraction patterns of the synthesized MoO_3_, Co_3_O_4_ and 10% Co_3_O_4_ modified MoO_3_ is presented in Figure 4. An orthorhombic phase of the synthesized α-MoO_3_ with lattice parameters ( a=3.9620 Å,b=13.8580 Å,c=3.6970 Å and α=β=γ=90° ) and cubic phase of Co_3_O_4_ having lattice parameters ( a=b=c=8.0840 Å and α=β=γ=90° ) was confirmed by matching diffractions patterns with ICDD# 00–006-0508 and 00–009-0418, respectively as shown in Figure 4A. The XRD pattern of 10% Co_3_O_4_ modified MoO_3_ in Figure 4A, clearly shows the dominancy of diffraction peaks arising due to the MoO_3_ as compared to the additional peaks due to the presence of cubic Co_3_O_4_ which have been marked by (*) in Figure 4B. The presence of additional peaks due to cubic Co_3_O_4_ in the XRD pattern of orthorhombic MoO_3_ confirms the composite nature of the newly synthesized photocatalysts. In addition to the presence of cubic Co_3_O_4_ (ICDD# 00–009-0418) as the major phase in α-MoO_3_, a couple of low-intensity reflections also specified the occurrence of monoclinic CoMoO_4_ (ICDD# 00–021-0868). The existence of CoMoO_4_ may be attributed to the attachment of Cobalt with the surface oxygen shared by Mo. The average crystallite sizes of the pristine MoO_3_ and 10% Co_3_O_4_ modified MoO_3_ were calculated using reflections at 2θ values of 12.68°, 23.27°, 25.87° and 27.25° with the help of the Debye–Scherrer equation. Wherein a mild increase in crystallite size for the composite sample was noticed as compared to pure MoO_3_. The estimated average crystallite sizes of α-MoO_3_, 10% Co_3_O_4_-MoO_3_ and Co_3_O_4_ powders were ∼54.42 nm, ∼58.43 nm and ∼20.63 nm, respectively. Average crystallite size of Co_3_O_4_ powder was evaluated using reflections at 2θ values of 31.25°, 36.84° and 65.22°.

**FIGURE 5:**
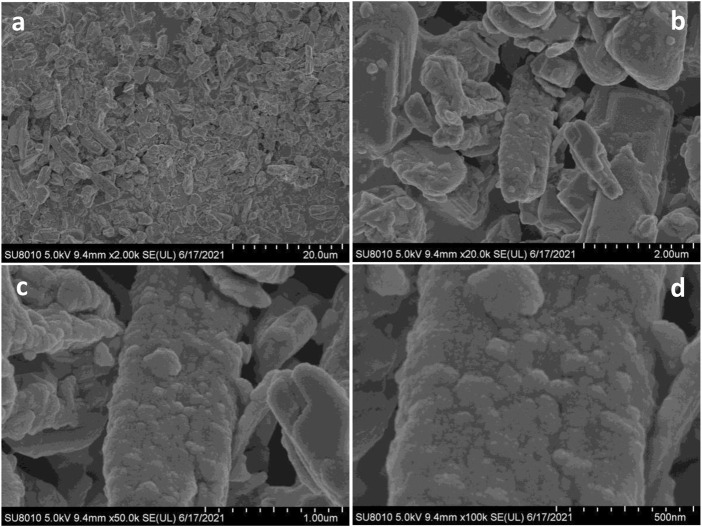
The scanning electron micrographs of 10% Co_3_O_4_-MoO_3_ at **(A)** 2.0 k **(B)** 20.0 k **(C)** 50.0 k and **(D)** 100 k magnifications.

Moreover, the morphology of 10% Co_3_O_4_-MoO_3_ was also explored at different magnification as shown in Figure 5 wherein the presence of Co_3_O_4_ were noticed on the surface of orthorhombic MoO_3_. The orthorhombic morphology of MoO_3_ was in accordance with XRD diffraction pattern of MoO_3_ (ICDD# 00–021-0868). The observed homogeneous distribution of Co_3_O_4_ on the surface of MoO_3_ verifies the composite form of the synthesized photocatalysts and the distributed Co_3_O_4_ played a significant role in enhancing the absorption of light and then increasing the photocatalytic efficiency of α- MoO_3_ in natural sunlight.

**FIGURE 6:**
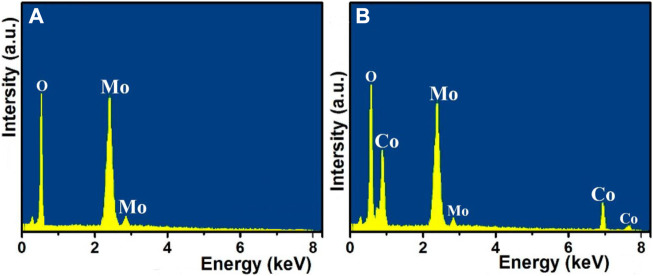
The EDX spectra of **(A)** pure MoO_3_ and **(B)** 10% Co_3_O_4_-MoO_3_.

Energy-dispersive X-ray spectroscopy was used to further determine the composition and element distribution of the MoO_3_ that had been synthesized in its as-prepared state (Figure 6A) and the 10% Co_3_O_4_-MoO_3_ (Figure 6B). According to the findings, which are displayed in Figure 6B, the 10% Co_3_O_4_-MoO_3_ composites displayed a spatial distribution of O, Mo, and Co species. The high intensity of the detection of O, Mo, and Co showed that each element was spread out evenly in the 10% Co_3_O_4_-MoO_3_ composites.

**FIGURE 7:**
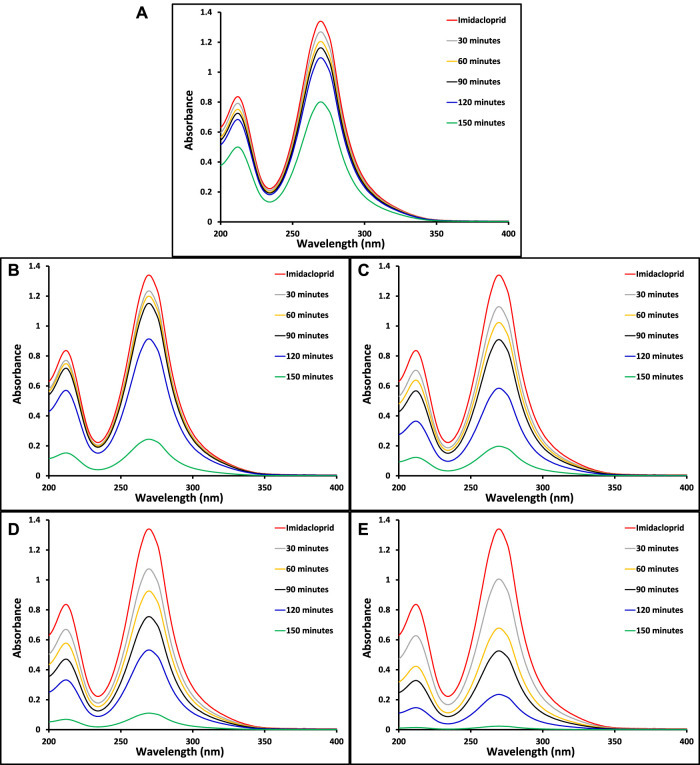
The comparison of absorption spectra for the removal of imidacloprid by **(A)** pristine MoO_3_ **(B)** 1% Co_3_O_4_-MoO_3_ **(C)** 3% Co_3_O_4_-MoO_3_ **(D)** 5% Co_3_O_4_-MoO_3_ and **(E)** 10% Co_3_O_4_-MoO_3_ at different light exposure time.

**FIGURE 8:**
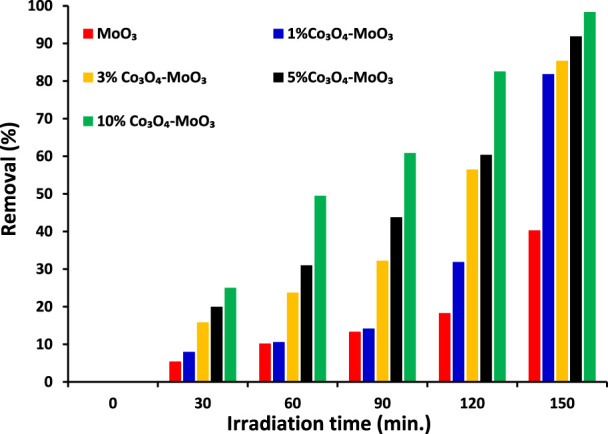
The comparison of removal (%) of 15 ppm imidacloprid by α-MoO_3_ and its composite with Co_3_O_4_ under the illumination of natural sunlight (800 ± 50 × 10^2^ lx) at different light exposures’ duration.

The ultimate goal of this study was to investigate the removal of imidacloprid and Greeda under the illumination of natural sunlight. Before the exposure of suspension containing photocatalyst and imidacloprid to light as mentioned in the experimental section, the suspension was kept in dark for 30 min to establish an equilibrium between pollutant and catalyst. The photolysis of imidacloprid was also evaluated by recording the absorption spectrum of the substrate after 150 min of light exposure without the presence of a photocatalyst. The amount of photocatalyst was also optimized (150 mg of photocatalyst) while studying the photocatalytic removal of 15 ppm imidacloprid with varying doses of MoO_3_ catalyst under the illumination of natural sunlight. The comparison of absorption spectra of imidacloprid’s removal over pristine and modified MoO_3_ under the illumination of sunlight (800 ± 50 × 102 lx) is provided in Figure 7 at different exposure times. Moreover, higher photodegradation (∼98%) was noticed for 10% Co3O4-MoO3 followed by 5% Co_3_O_4_-MoO_3_ (∼92%), 3% Co_3_O_4_-MoO_3_ (∼85%) and 1% Co_3_O_4_-MoO_3_ (∼81%) and MoO_3_ (∼41%) after 150 min of sunlight exposure. As shown in Figure 8 The decreasing trend of removal efficiency (%) of the photocatalysts is given below.

10 % Co_3_O_4_-MoO_3_> 5 % Co_3_O_4_-MoO_3_ > 3 % Co_3_O_4_-MoO_3_ > 1 % Co_3_O_4_-MoO_3_ > MoO_3_

**FIGURE 9:**
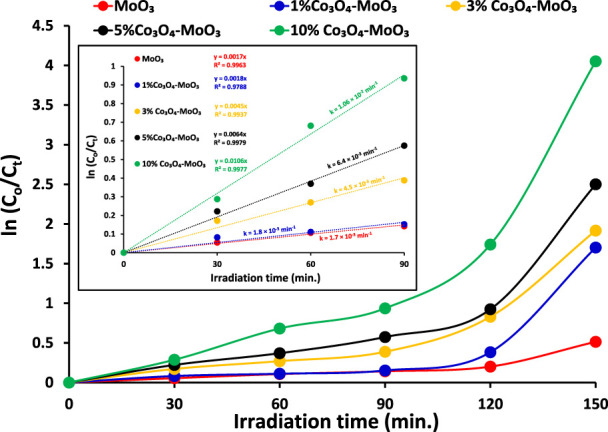
The comparison of rate of removal of imidacloprid (15 ppm) by α-MoO_3_ and its composite with Co_3_O_4_ under the illumination of natural sunlight (800 ± 50 × 10^2^ lx). Whereas, inset shows the estimation of rate constants.

**FIGURE 10:**
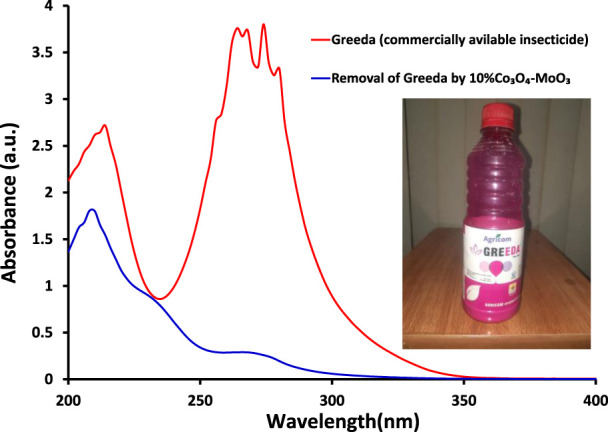
The comparison of absorption spectra of Greeda, a commercially available insecticide, (red) initially taken and (blue) after 150 min of light exposure in the presence of 10% Co_3_O_4_-MoO_3_ photocatalyst.

**FIGURE 11:**
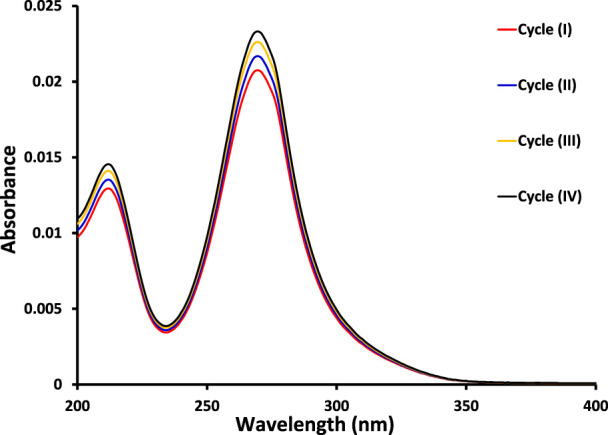
The comparison of absorption spectra for the removal of imidacloprid by 10% Co_3_O_4_-MoO_3_ photocatalyst in exposure of natural sunlight for 150 min in four cycles at an interval of 24 h.

**SCHEME 3:**
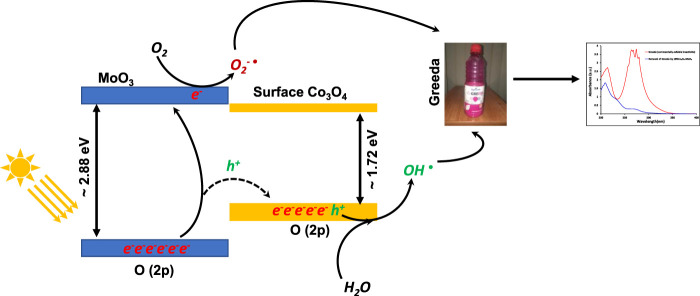
The schematic representation for the absorption of photon, photo-excitons’ separation and the removal of Greeda in the exposure of natural sunlight.

The kinetic study reveals that the photocatalytic removal of imidacloprid by newly synthesized materials did not follow the Langmuir Hinshelwood (L-H) kinetic model throughout light exposure from 0 to 150 min as shown in Figure 9. However, in the initial 90 min the rate of removal followed Langmuir Hinshelwood (L-H) kinetic model and the estimated rate constants are 1.7 × 10^−3^ min^-1^, 1.8 × 10^−3^ min^-1^, 4.5 × 10^−3^ min^-1^, 6.4 × 10^−3^ min^-1^ and 1.1 × 10^−2^ min^-1^ by MoO_3_, 1% Co_3_O_4_-MoO_3_, 3% Co_3_O_4_-MoO_3_, 5% Co_3_O_4_-MoO_3_ and 10% Co_3_O_4_-MoO_3_, respectively as provided in the inset of Figure 9. The higher removal efficiency of Co_3_O_4_-MoO_3_ as compared to pristine MoO_3_ may be attributed to the synergic role of Co_3_O_4_ in increasing the life span of photo-excitons by lowering the charge recombination process (Figure 3), which ultimately leads to the higher removal of imidacloprid. In order to assess the application of an efficient photocatalyst declared in this study, i.e., 10% Co_3_O_4_-MoO_3_ for the removal of insecticide, Greeda (a commercially available frequently applied insecticide in the agriculture field) was chosen. Figure 10 shows that ∼93% of Greeda was removed by 10% Co_3_O_4_-MoO_3_ in 150 min of natural sunlight exposure. Previously, the photocatalytic removal of imidacloprid was explored previously using iron-based catalysts, ZnO, TiO_2_, SnO_2_ ternary nanocomposites, g-C_3_N_4_ and WO_3_ under the exposure of UV and visible light, however, studies pertaining to the removal of Greeda and Imidacloprid using Co_3_O_4_-MoO_3_ photocatalysts are not evident from the literature. (Guzsvány et al., 2010; Derbalah et al., 2019; Yari et al., 2019; Sudhaik et al., 2020; Li et al., 2022; Faisal et al., 2023). The efficiency of 10% Co_3_O_4_-MoO_3_ photocatalyst after four cycles was also investigated and a minor decrease in efficiency was noticed for the removal of imidacloprid as shown in Figure 11. Moreover, the mechanism for the removal of Greeda in the exposure of natural sunlight by Co_3_O_4_ modified MoO_3_ composite is provided in Scheme 3.

## Conclusion

The successful synthesis of Co_3_O_4_-modified MoO_3_ composites through the wet-impregnation method and their photocatalytic activities lead to the following conclusions.

• The successfully distributed Co_3_O_4_ on the surface of orthorhombic MoO_3_ played a significant role in improving the spectral response and lowering the bandgap energy of α- MoO_3_ from ∼2.88 eV to ∼2.15 eV.
• The higher *e* ^*-*^–*h* ^*+*^ recombination in α- MoO_3_ was successfully suppressed by modifying MoO_3_ with Co_3_O_4_.
• All modified MoO_3_ photocatalysts presented higher photocatalytic activity for the removal of imidacloprid under the illumination of natural sunlight due to the synergic effect of Co_3_O_4_.
• A commercially available insecticide (Greeda) was successfully removed (∼93%) by 10% Co_3_O_4_-MoO_3_ in 150 min of natural sunlight exposure.

## Data Availability

The original contributions presented in the study are included in the article/supplementary material, further inquiries can be directed to the corresponding authors.
